# Berlin Heart bridge to transplantation in an 18-month-old with septicaemia and thrombocytopenia: a case report and literature review

**DOI:** 10.1007/s12055-025-01994-9

**Published:** 2025-08-06

**Authors:** Ashmit Bhardwaj, Rishabh Suvarna, Deepa Sarkar, Reetesh Gupta, Bhaba Nanda Das, Jothi Muthu, Mukesh Goel

**Affiliations:** 1 School of Medicine, University of Leeds, Worsley Building, Woodhouse, Leeds, LS2 9 JT UK; 2 Department of Cardiac Surgery, Indraprastha Apollo Hospital, New Delhi, Delhi 110076 India; 3 Department of Paediatric Cardiac Intensive Care, Indraprastha Apollo Hospital, New Delhi, Delhi 110076 India; 4 Department of Cardiac Anaesthesia, Indraprastha Apollo Hospital, New Delhi, Delhi 110076 India

**Keywords:** Berlin Heart EXCOR®, Paediatric ventricular assist device, Dilated cardiomyopathy, Heart failure, Heart transplantation

## Abstract

Dilated cardiomyopathy (DCMP), a non-ischaemic heart disease involving ventricular enlargement, is the second most common cause of heart failure, with a prevalence of 1:2500. Few studies document the use of paediatric ventricular assist devices (VADs) such as the Berlin Heart EXCOR (BHE) device in those with sepsis. We report an 18-month-old patient, diagnosed with DCMP-induced refractory heart failure, causing septicaemia and thrombocytopenia. Despite this, BHE implantation was used as a last resort bridge to heart transplantation. Our case highlights the importance of carefully managing BHE in high-risk, compassionate-use patients, challenging existing guidelines and encouraging individualised assessments in VAD application.

## Introduction

Dilated cardiomyopathy (DCMP) is a non-ischaemic disease of the heart, characterised by biventricular or univentricular enlargement and dilatation of the myocardium. A diagnostic hallmark of this condition is impaired contractility in the absence of coronary artery disease (left ventricular ejection fraction (LVEF) < 45% and or fractional shortening < 25%), notably of the left ventricle. DCMP is therefore one of the commonest causes of heart failure, necessitating cardiac transplantation. Due to their size and age, paediatric donor hearts are scarce, prolonging waiting times.

Ventricular assist devices (VADs) are hence used as a bridging therapy until suitable donor hearts are available. Berlin Heart EXCOR (BHE), a pneumatically powered, para-corporeal, pulsatile flow VAD, is the most common paediatric VAD utilised globally [1].

We report the case of an 18-month-old girl with sepsis secondary to DCMP-induced heart failure, who was managed with the BHE device, alleviating the septicaemia and enabling heart transplantation. To our knowledge, this is the first report documenting the fine details of an unusual case of BHE implantation, despite severe septicaemia and thrombocytopenia, contraindicated by existing guidelines.

## Case report

An 18-month-old girl presented in the casualty ward with significant breathlessness. The patient went into cardiac arrest; thus, cardiopulmonary resuscitation (CPR) was commenced for 20 min. Other than sinus tachycardia, the patient’s electrocardiogram (ECG) was normal. The patient previously had several episodes of heart failure and was consequently placed on the transplant list. An echocardiogram revealed moderate mitral regurgitation and an LVEF of 15%.

**Fig. 1:**
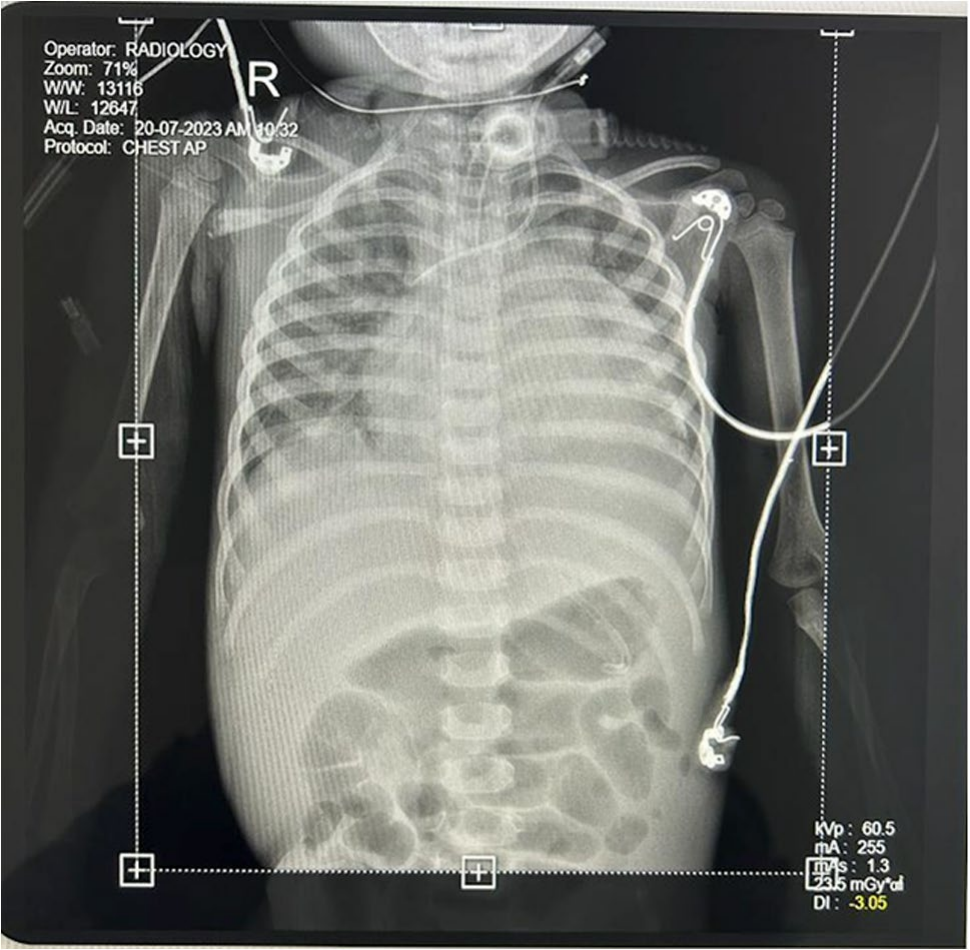
Anterior–posterior (AP) chest X-ray of the patient with dilated cardiomyopathy (DCMP)

A chest X-ray revealed cardiomegaly, showing an enlarged, globular-shaped heart (Fig. 1). Altogether, the above findings were consistent with DCMP. She was on triple inotropes (milrinone, dobutamine, adrenaline), diuretics, and mechanical ventilator support. Unable to wean her off inotropes and the ventilator, a tracheostomy was performed in view of prolonged ventilatory support.

This was complicated by septicaemia, triggering disseminated intravascular coagulation (DIC) and causing thrombocytopenia (platelet count = 17,000/µL). The patient had raised bilirubin due to septicaemia-induced cholestasis. This necessitated immediate management; however, the septicaemia was unresponsive to broad-spectrum antibiotics and antifungals.

**Fig. 2:**
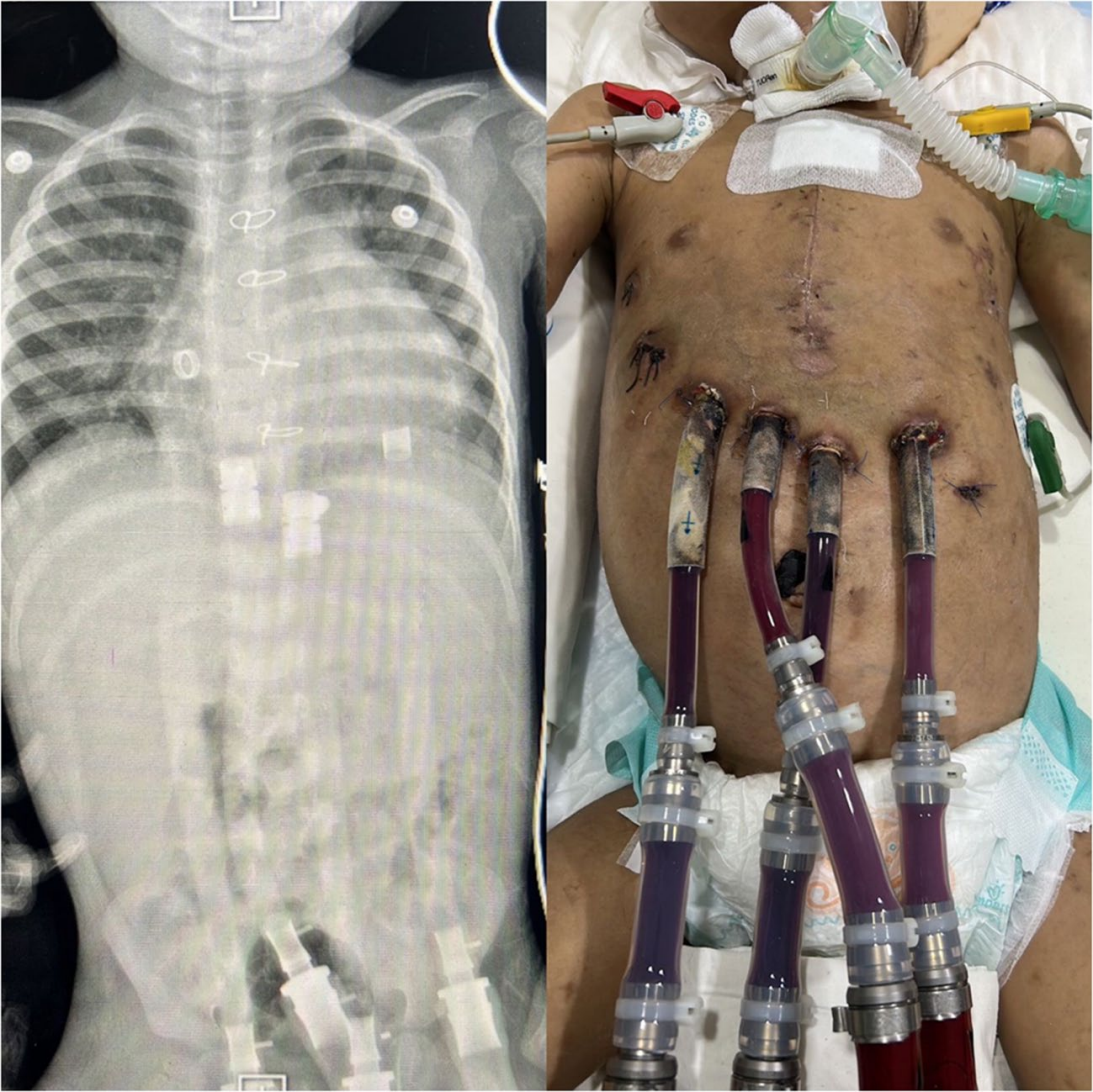
Berlin Heart implantation (with X-ray showing the drivelines)

Due to refractory heart failure complicated by septicaemia, thrombocytopenia, and hyperbilirubinemia, BHE implantation was considered as a last resort until donors were available (Fig. 2).

Two months post-admission and within days of triple inotrope therapy failure, the BHE was implanted, alongside anticoagulation. During BHE transplantation, a biopsy taken from the myometrial tissue of the left ventricle revealed focal mild inflammation composed of scattered lymphocytes, mild reactive nuclear atypia of the myocytes, scalloped appearance of myocytes, interstitial oedema, hydropic degeneration, and mild architectural distortion.

Post-BHE implantation, along with adjuvant therapies of inotropes, antibiotics, and antifungals, severe septicaemia, abdominal distension, and peripheral oedema quickly resolved. The patient was weaned off the ventilator, and inotropes were tapered within 7 days. The tracheostomy was removed in 4 weeks, and she was actively mobilised. A month later, the patient suffered a minor stroke, which was managed with increased warfarin. Three months later, a donor heart was found and transplanted successfully.

Following this, the excised heart was sent to histopathology and demonstrated dilatation of all chambers, focal yellowish gritty areas in the myocardium of the ventricles, and a yellowish mural thrombus in the right ventricular cavity. Multiple sections from the walls of both atria and ventricles revealed an expanded endocardium, showing fibrosis and focal myxoid degeneration.

Six weeks post-transplantation, she was discharged with maintenance doses of tacrolimus, azathioprine, and prednisolone as immunosuppression. Six months post-discharge, prednisolone was weaned off, and the patient underwent rehabilitation and now continues to thrive, with normal donor-derived cell-free Deoxyribose Nucleic Acid (DD-cfDNA) and endomyocardial biopsy confirming no signs of rejection.

## Discussion

For years, extracorporeal membrane oxygenation (ECMO) was commonly used as a bridging therapy to cardiac transplantation due to the lack of Food and Drug Administration (FDA)–approved VADs designed specifically for the paediatric population, despite its poor success. However, since its inception, BHE has been consistently demonstrated to significantly increase survival time to transplant and long-term survival rates without a significant increase in stroke or multisystem organ failure [2]. It is now the most widely used mechanical circulatory support system designed for infants and children of body surface area ≤ 0.6 m^2^ with heart failure, with the majority of the indications for DCMP [3].

Despite this, recent systematic reviews have highlighted serious complications with its use, most commonly ischaemic stroke and pump thrombosis [4, 5]. This arises due to valvular thrombosis on the BHE device [6], occurring early in the course of treatment and being the most prominent cause of neurological injury and mortality [7]. However, strict anticoagulation regimens have demonstrated success in reducing thrombosis-related stroke incidence by up to 84% compared to previous protocols [8].

Unfortunately, there is still a paucity of precise and up-to-date guidelines for BHE implantation in children, despite many advancements in this field. According to FDA Guidelines, BHE is usually contraindicated in children unable to sustain systemic anticoagulation, potentially excluding a large subset of the target population, as epidemiological studies have demonstrated that paediatric patients with pre-existing cardiomyopathies are at greater risk of septicaemia and hence DIC [9]. This is further supported by the observation that only one-fourth of all potential participants were captured within the FDA trial. However, our case illustrates that septicaemia and thrombocytopenia are not absolute contraindications to BHE therapy, showing that it should be considered as an option when medical therapies have been exhausted in paediatric patients with heart failure, given the lack of alternative options providing comparable survival likelihoods.

This is further evident with the compassionate cases from the FDA trial, illustrating survival rates of 75% and comparable risk of stroke in this population to eligible patients [10]; however, this included patients with other exclusion criteria (e.g. complex heart disease, significant end-organ dysfunction pre-implantation). Hence, risk factors specific to each exclusion criterion, such as inability to sustain systemic anticoagulation, should be further investigated in large-scale cohort studies to provide stronger evidence and enable better decision-making in such scenarios.

Overall, our case demonstrates the importance of careful patient selection and timing in BHE implantation, providing guidance on successful BHE implantation and management in such complex cases. BHE remains an effective bridging therapy in patients with cardiomyopathies requiring heart transplantation, but implantation must be completed prior to significant multi-organ failure and timely enough to avoid unnecessary exposure to the risks of BHE. In cases where patients possess known mortality risk factors for BHE implantation, patients should be closely monitored, and strict anticoagulation protocols should be applied to avoid thrombotic complications.

## Conclusion

This case highlights the use of the BHE as a bridging therapy to heart transplantation in a high-risk paediatric patient with refractory dilated cardiomyopathy complicated by severe septicaemia and thrombocytopenia. While existing contraindications suggest caution, we emphasise that in specific, critically ill patients where all other medical therapies have failed, timely BHE implantation—combined with vigilant anticoagulation and multidisciplinary care—can enable recovery from life-threatening complications and facilitate successful heart transplantation.

Our findings underscore the importance of individualised patient assessment and a nuanced risk–benefit analysis in complex cases, suggesting that under certain circumstances, traditional contraindications to BHE implantation may be challenged. The successful outcome highlights the potential of BHE to improve survival and quality of life for paediatric patients with severe cardiac failure, even in the presence of high-risk factors. Future research should focus on refining guidelines and exploring large-scale cohort studies to better understand risk mitigation strategies and optimise decision-making in similar scenarios. This case exemplifies the critical role of compassionate, innovative approaches in advancing paediatric cardiac care.

## Data Availability

Anonymized data shall be provided on reasonable request.
